# Managing the Pandemic: The Italian Strategy for Fighting COVID-19 and the Challenge of Sharing Administrative Powers

**DOI:** 10.1017/err.2020.82

**Published:** 2020-09-03

**Authors:** Donato VESE

**Affiliations:** * University of Turin, Department of Law, Turin, Italy and University of Oxford, Faculty of Law, Centre for Socio-Legal Studies, Oxford, UK; email: donato.vese@unito.it/donato.vese@csls.ox.ac.uk.

## Abstract

This article analyses the administrative measures and, more specifically, the administrative strategy implemented in the immediacy of the emergency by the Italian government in order to determine whether it was effective in managing the COVID-19 pandemic throughout the country. In analysing the administrative strategy, the article emphasises the role that the current system of constitutional separation of powers plays in emergency management and how this system can impact health risk assessment. An explanation of the risk management system in Italian and European Union (EU) law is provided and the following key legal issues are addressed: (1) the notion and features of emergency risk regulation from a pandemic perspective, distinguishing between risk and emergency; (2) the potential and limits of the precautionary principle in EU law; and (3) the Italian constitutional scenario with respect to the main provisions regulating central government, regional and local powers. Specifically, this article argues that the administrative strategy for effectively implementing emergency risk regulation based on an adequate and correct risk assessment requires “power sharing” across the different levels of government with the participation of all of the institutional actors involved in the decision-making process: Government, Regions and local authorities.

“And the flames of the tripods expired. And Darkness and Decay and the Red Death held illimitable dominion over all”.

Edgar Allan Poe, *The Mask of the Red Death, Complete Tales and Poems*

(New York, Vintage Books 1975) p 273

## I. Introduction

COVID-19 has become a global health emergency.^1^ The World Health Organization (WHO) initially declared the COVID-19 outbreak a “Public Health Emergency of International Concern” (PHEIC).^2^ In the light of its later levels of spread and severity worldwide, the WHO then assessed COVID-19 as a “pandemic”.^3^

The pandemic has spread rapidly in several European Union (EU) Member States. Italy, however, is a special case: here, the COVID-19 outbreak spiralled upwards earlier and more severely than elsewhere in Europe, reaching a high mortality rate and creating the conditions for the public healthcare system’s collapse.

In this scenario, the Italian government (from now on the Government) declared a nationwide state of emergency,^4^ followed by increasingly restrictive measures aimed at slowing and containing the spread of the virus and mitigating the pandemic’s effects under the by now well-known “flatten the curve” imperative. The last of these measures^5^ established the national lockdown, extending the emergency rules to the entire country for six months^6^ and, more generally, providing what has been called the “Italian model to fight COVID-19”, namely “diminish viral contagions through quarantine; increase the capacity of medical facilities; and adopt social and financial recovery packages to address the pandemic-induced economic crisis”.^7^

In this article, starting from the main regulatory acts and considering recent scientific knowledge and epidemiological data on COVID-19, we will examine the administrative measures the Government has taken and the strategy it has implemented to deal with the pandemic in the immediacy of the emergency. After this initial analysis, we might legitimately wonder whether those measures and that strategy have proven effective in containing the pandemic.

More generally, by analysing the administrative strategy, the article emphasises the role that the current system of constitutional separation of powers plays in emergency management and how this system can impact health risk assessment.

An explanation of the risk-management system in Italian and EU law will be provided and the following key legal issues will be analysed: (1) the notion and features of emergency risk regulation from a pandemic perspective, distinguishing between risk and emergency; (2) the potential and limits of the precautionary principle in EU law; and (3) the Italian constitutional scenario with respect to the main provisions regulating central government, regional and local powers.

Specifically, the article argues that the administrative strategy for effectively implementing emergency risk regulation based on an adequate and correct risk assessment requires “power sharing” across the different levels of government with the participation of all of the institutional actors involved in the decision-making process: Government, Regions and local authorities.

## II. The strong strategy: the emergency measures for pandemic management

Following the declaration of the state of emergency, the Government approved Decree-Law No. 6 of 23 February 2020 vesting the President of the Council of Ministers with wide ordinance powers to handle the emergency by issuing his own administrative decrees.^8^

In particular, Decree-Law 6/2020 gave the Prime Minister the power to issue typical emergency administrative measures in order to ensure social distancing, impose lockdown areas, close offices and public services and suspend economic activities. In addition, it allowed him to adopt atypical administrative powers whereby “further containment and emergency management measures” could be established.^9^

In a matter of days, the Government approved three important regulatory acts based on the implementation of Decree-Law 6/2020:^10^ first with the Decree of the President of the Council of Ministers (DPCM) of 8 March 2020,^11^ second with the DPCM of 9 March 2020^12^ and third with the DPCM of 11 March,^13^ the Government established stringent emergency administrative measures to curb the pandemic’s spread throughout the country.^14^

In the first instance, these measures were gradual and concerned specific municipalities, provinces or regions – especially in northern Italy – that were hardest hit by the virus and therefore classified as “red zones” subject to Government-imposed local lockdowns. Later on, the Government established the national lockdown, and emergency measures were extended to the entire country for six months.

In particular, pursuant to Article 1(1) of the DPCM of 8 March 2020, the Government imposed a lockdown in Lombardy and another fourteen provinces of northern Italy. In doing so, the Government introduced several legal prohibitions, such as the ban on people travelling to and from places in the red zones. With the subsequent national lockdown, the Government imposed a travel ban in the entire country according to Article 1(1), DPCM of 9 March 2020, and prevented all forms of social gathering in public places or places open to the public across the country, according to Article 1(2), DPCM of 9 March 2020. Furthermore, pursuant to Articles 1(1), 1(2) and 1(3), DPCM of 11 March 2020, retail businesses and personal services were suspended.^15^

As a consequence of the national lockdown, the Ministry of Health’s Order of 20 March 2020 provided several stringent measures that prohibited many activities, such as the ban on accessing all public places, on exercising in public places and on going to holiday homes.^16^ In addition, with its order of 28 March 2020, the Ministry of Health, in agreement with the Ministry of Transport, established that people entering Italy by plane, boat, rail or road must declare their reason for travel, the address where they plan to self-isolate, how they intend to travel there and their phone number so that authorities can contact them throughout an obligatory fourteen-day quarantine.^17^

Moreover, several administrative sanctions were gradually established in the various regulatory acts. The last of these acts introduced rigorous sanctions for people who leave home without valid reasons and for undertakings that do not comply with the order to close.^18^

In the meantime, the Regions and local authorities also adopted several ordinances establishing emergency administrative measures for the pandemic in their area.^19^ Lastly, the Government issued Decree-Law No. 19 of 25 March 2020, with the aim of rationalising and coordinating emergency powers among the different levels of government.^20^

***

In the following pages, emphasising the role that the current structure of constitutional separation of powers plays in risk assessment, I will argue that the main problems of the Italian administrative strategy for the COVID-19 pandemic are due to the lack of effective “sharing of powers”, and more specifically to the failure to share administrative regulatory powers among the different levels of government with the participation and cooperation of all institutional actors involved in the emergency decision-making process: the Government, Regions and local authorities.^21^

From this point of view, as I will attempt to explain, the failure to share administrative regulatory powers can have a decisive impact on risk assessment at the national level in terms of the effectiveness/ineffectiveness of the strategies adopted by the various institutional actors called upon to manage the emergency in their own areas.

Here, by “sharing powers”, I mean the idea that the institutional actors involved in the decision-making process cooperate in the exercise of their powers by adopting consistent measures in the public interest; that is to say, with the aim of maximising the rights of individuals as required by the Italian Constitution.^22^

Power sharing does not mean homologation. Indeed, adopting different administrative strategies at different levels of government might increase the effectiveness of the response to a pandemic, but these measures must be shared among all of the actors involved in emergency management.

Sharing powers, measures and local strategies will be useful for an effective policy for containing the virus’s nationwide spread based on an overall risk assessment.

Hence, the idea of shared powers emphasises the role of cooperation in specific institutional contexts, such as Italy’s, where competences are allocated across the different levels of government. The sense, more generally, is that sharing powers in multi-level systems enables States to perform better in terms of democracy, as powers are balanced between state and local levels.^23^

As we will see, however, the absence of effective power sharing at all levels of government in a pandemic can produce serious problems in correctly assessing risk and consequently in the emergency management strategy.

In particular, I will discuss the problem of the lack of effective power sharing in Italian policies from two key points of view: the Government’s administrative strategy in addressing the virus’s spread by means of an “incremental approach” (Section IV.1.a); and the Government’s administrative strategy in implementing a national pandemic health plan (Section IV.1.b).

Before doing so, I will outline some key legal issues for the topics examined in this article. In particular, to put the administrative strategy devised by the Government in the COVID-19 emergency into context, I will analyse: (1) the notion and features of emergency risk regulation from a pandemic perspective, distinguishing between risk and emergency; (2) the potential and limits of the precautionary principle in EU law; and (3) the Italian constitutional scenario with respect to the main provisions governing government’s, regions’ and local authorities’ powers.

This preliminary analysis of key legal issues is useful for understanding why the administrative strategy has proven ineffective in managing the pandemic (Sections IV.1.a and IV.1.b).

## III. Key legal issues in the context of the pandemic

### 1. Emergency risk regulation

Placing the notion and its main features in the context of a pandemic, we could define emergency risk regulation as the action undertaken in the immediacy of a pandemic in order to mitigate its impact.^24^

From this perspective, we should bear in mind the distinction between risk and emergency. Generally speaking, the traditional approach of administrative law refers to the notion of emergency and not also to the notion of risk, which legal doctrine touches on only marginally.^25^ With regards to the emergency, as a safeguard clause to deal flexibly with pandemic risks,^26^ governments and other public authorities may invoke the use of extraordinary powers to restore the normal course of legal relations.^27^ What is more, regulators have used emergency tools to act in the expectation of a risk for many years, although there is no denying that a risk is a potential danger, whereas an emergency is an actual danger. Indeed, it should be sufficiently clear that emergency power is ineffective when applied in a situation that is only potentially dangerous. In this connection, it has been argued^28^ that the methods of exercising administrative powers can be better regulated by putting the administrative regulation in the category of risk rather than that of emergency.

We might observe that if the notion of “risk” characterises a peculiar, intermediate state between security and destruction,^29^ in “emergency risk” the balance between these two clearly tilts towards the latter.^30^ In fact, as it is triggered by a pandemic, emergency risk regulation presupposes the existence, or the mere threat, of a pandemic. The pandemic, as a possible cause of disaster for humans, is an event of substantial extent causing significant physical damage or destruction, loss of life or drastic change to the natural environment.^31^ Typically, one speaks of a pandemic when a threat to people’s health is perceived that calls for urgent remedial action under conditions of uncertainty.^32^

Fundamentally, emergency risk regulation in a pandemic event, as in other disasters, finds its natural regulatory space in two stages: mitigation and emergency response.^33^ In principle, mitigation efforts attempt to reduce the potential impact of a pandemic before it strikes, while a pandemic response tends to do so after the event. However, the distinction between emergency mitigation and emergency response is not always very sharp. When called upon to act under the menace of a pandemic, governments must both mitigate and respond to the threat in a situation characterised by suddenness (emergency) and significance.^34^ In a pandemic, emergency risk regulation is clearly called on to operate in the initial phase of the disease’s spread, when the mere threat overshadows the regulatory context by virtue of its status as an emergency.

Accordingly, the most cost-effective strategies for increasing pandemic preparedness with administrative regulation, especially in resource-constrained settings, may consist of: (1) investing to reinforce the main public health infrastructure; (2) increasing situational awareness; and (3) quickly containing further outbreaks that could extend the pandemic. In addition, especially once the pandemic has begun, a coordinated response should be implemented where the public regulator focuses on: (4) maintaining situational awareness; (5) public health messaging; (6) reducing disease transmission; and (7) care and treatment of the ill. Successful contingency planning and an administrative strategy using the emergency risk regulation approach call for surge capacity, or in other words the ability to scale up the delivery of health interventions in proportion to the severity of the event, the pathogen and the population at risk.^35^

The pandemic may produce significant impact on the regulatory context by justifying the partial or total suspension of the ordinary decision-making process.^36^ Departures from the rule of law, or simply from established procedures, are generally perceived as necessary if the event has met the significance threshold. However, the use of emergency administrative measures, such as temporary and exceptional measures, should be considered legitimate only for the period in which the pandemic lasts.^37^ By contrast, prolonging exceptional order beyond the time of the pandemic means that any powers and measures designed to be temporary will be made permanent, intensifying the controlling authority’s capacity, even though this might limit the enjoyment of individual rights.^38^ In addition, if the general need to prevent a pandemic cannot be ignored, it should be well thought out as an opportunity for risk regulation to prevent not only the sudden impact of a pandemic situation, but also any distorting effects or mishandling of the necessary recourse to emergency powers.

Consequently, it might now be inferred that emergency risk regulation in the context of a pandemic is a relevant regulatory methodology that combines the risk approach with the possibility of resorting to extraordinary measures in case a pandemic occurs. This methodology is essential for an effective administrative strategy for dealing with a pandemic because it permits constant monitoring and management of risks that can have serious consequences for society. By assessing the risks and taking proportionate measures, the negative effects of the emergency can be reduced and the use of emergency powers can be limited.

Indeed, it should be pointed out that the principle of reasonableness, which is generally invoked in the exercise of emergency powers against immediate danger, does not operate in emergency risk regulation. Instead, as I will claim later, it will be the precautionary principle that matters (Section III.2). Furthermore, it must be said that emergency risk regulation entails an accurate assessment of the factual situation based on scientific evidence.^39^ To apply this methodology correctly, a variety of factors must be considered – including the real level of the threat as well as how people perceive it – in a step-by-step analysis based on the available scientific knowledge.

In particular, as I will claim in analysing the Italian policies (Sections IV.1.a and IV.1.b), the administrative strategy for effectively implementing emergency risk regulation in a pandemic requires power sharing across the different levels of government with the participation of all of the institutional actors involved in the decision-making process in order to adopt consistent measures based on the constant monitoring and updating of the nationwide epidemiological risk assessment.

Hence, effective sharing of administrative powers – and more specifically the administrative regulatory powers for emergencies – between the Government, Regions and local authorities would optimise the adoption of proportionate measures for controlling and containing the virus throughout the country, avoiding or at least delaying the application of stringent measures such as the lockdown of municipalities, provinces, regions or entire states.

In managing the pandemic, the Government’s administrative strategy should take the emergency risk regulation methodology we have just outlined into account.

### 2. The precautionary principle in EU law: potential and limits

In the EU legal system, the precautionary principle^40^ is described in Article 191(2) TFEU on environmental policy.^41^

The jurisprudence of the European Court of Justice (ECJ) played a prominent role in elevating the precautionary principle to the status of a general principle of EU law. Some ECJ judgments in health matters are seminal in this regard.^42^ According to the ECJ’s jurisprudence, the precautionary principle requires that competent authorities adopt appropriate administrative measures to prevent specific potential health risks. The ECJ’s approach maintains that an appropriate application of the precautionary principle presupposes the identification of hypothetically harmful effects for health flowing from the contested administrative measure, combined with comprehensive assessment of the risks to health based on the most reliable scientific data available.^43^

In like manner, the European Commission (EC) has contributed significantly to outlining the features of the precautionary principle in the EU legal system. In the Communication of 2000, the EC sought to establish a common understanding of the factors leading to recourse to the precautionary principle and its place in decision-making.^44^ According to the EC Communication, the principle covers those circumstances where scientific evidence is insufficient, inconclusive or uncertain, but where preliminary scientific evaluation provides reasonable grounds for concern that the potentially dangerous effects on human health might be inconsistent with the chosen level of protection.^45^ Various factors can trigger the adoption of precautionary measures. These factors inform the decision on whether to act or not, this being an eminently political decision, a function of the risk level that is “acceptable” to the society on which the risk is imposed.^46^

The EC has also established guidelines for those situations where action based on the precautionary principle is deemed necessary in order to manage risk. In these situations, a cost–benefit analysis to compare the likely positive and negative effects of the envisaged action and of inaction is recommended, and it should also include non-economic considerations.^47^ However, risk management in accordance with the precautionary principle should be proportionate, meaning that administrative measures should be proportional to the desired level of protection. In some cases, an administrative response that imposes a total ban may not be proportional to a potential risk; in others, it may be the only possible response. In any case, such measures should be reassessed in the light of recent scientific data and changed if necessary.

In EU law, therefore, the precautionary principle has been widely recognised as a defining principle of risk regulation alongside the regulatory aim of a high level of protection. Nevertheless, this principle might prove ineffective or even harmful if applied in a “strong” form. The strong form of the principle has been authoritatively criticised^48^ on the grounds that it suggests that regulation is required whenever there is a potential risk to health, even if the supporting evidence is conjectural and the economic costs of administrative regulation are high.

In particular, if governments adopt the strong form of the principle, it would always require regulating activities – consequently imposing a burden of proof each time – even if it cannot be demonstrated that those activities are likely to cause harms.^49^ In addition, as the need for selectivity of precautions is not simply an empirical fact but is a conceptual inevitability, no society can be highly precautionary with respect to all risks.^50^

Hence, in this strong form, the precautionary principle proves ineffective and even harmful by requiring stringent administrative measures that can be paralysing, in that they prohibit regulation and all courses of action, including inaction. Thus conceived, this principle may not lead in any direction or provide precise guidance for governments and regulators.

Recently, the limits of the precautionary principle have been analysed in the field of administrative and constitutional law. An interesting recent work proposes that precautionary and optimising constitutionalism are a dichotomy.^51^ In summary, the theory advances two distinct propositions. The first is that constitutions should be viewed as devices for regulating political risks. Those political risks are referred to as “second-order risks”, as opposed to “first-order risks” such as wars, diseases and other social ills.^52^ Many of these risks are described as “fat-tail risks” that are exceedingly unlikely to materialise, but more likely than in a normal distribution, and are exceedingly damaging if they do materialise, as in the case of a pandemic.^53^ Under “maximin constitutional” approaches, it is suggested that precautionary rules can overcompensate for these low-likelihood risks and even cause the very dangers that they seek to prevent.^54^ Hence, precautionary constitutionalism is myopic in focusing on certain risks, and the notion of unappreciated or unaccommodated risks is central. On the basis of this hypothesis, the best way to regulate risk is thus to avoid obsessive views on risk avoidance or precautions and instead to allow greater flexibility in addressing the full array of risks inherent in government.^55^ What Vermeule calls “optimising constitutionalism” is an answer to those who frame their understanding of the Constitution along more rigid precautionary principles.^56^

Vermeule’s approach has been criticised.^57^ Following these criticisms, I believe that this approach also reveals some critical points about the notion of risk. Unless one adopts a more fungible notion of risk, I do not believe that “precautionary constitutionalism” is suboptimal for risk. It depends on how one weighs the risks involved in governing, even if one accepts risk analysis as the best measure for the success of a constitutional system.

I claim, more generally, that correctly applying the precautionary principle, although it works better in a context of risk rather than one of emergency, is nonetheless important in managing a pandemic because it makes it possible to delay the implementation of stringent emergency measures. We have emphasised that administrative precautionary measures, unlike emergency ones, do not suspend the rule of law, since they activate soft government regulation that does not jeopardise fundamental rights concurrent with those threatened by imminent danger.

Hence, in my opinion, precautionary measures, where they are effectively shared across the different levels of government through appropriate risk assessment, would serve to avoid or at least delay governments’ activation of a state of emergency. Activating a state of emergency, consequently, would trigger hard government regulation through emergency measures that suspend the rule of law and therefore jeopardise fundamental rights.

In a particular context such as the COVID-19 pandemic, the precautionary principle could also be invoked – and the implementation of precautionary administrative measures would be useful – in the presence of an emergency declaration issued by governments. In this sense, I argue that the declaration of a state of emergency for a pandemic is based on a technical risk assessment (ie technical discretion^58^) by the administration (eg government). In a pandemic, then, the emergency relates essentially to the capacity of administrations (eg governments, health authorities) to manage cases requiring healthcare (eg intensive care for respiratory support, hospitalisations for advanced pharmacological treatments and so on). Thus, the subject of the technical assessment of the fact (the pandemic) is be provided by the evaluation relating to the administration’s capacity to fulfil the tasks established by the legal system to protect the right to health enshrined in Article 32 of the Italian Constitution (Section IV.2).

Furthermore, to be effective in emergencies such as a pandemic, the notion of the principle to which I refer should not entail the activation of precautionary measures typical of its strong version (which is exemplified in the well-known phrase “better safe than sorry”). In its strong version, in fact, the precautionary principle would be both paralysing and uneconomical, since it requires that any and all risks be prevented, even those that are least likely to occur or have been created artificially for political reasons (I am thinking here of George W. Bush’s preventative war doctrine) in order to justify stringent administrative measures issued by governments for purposes not necessarily related to the alleged risk.

By contrast, balancing costs against benefits might provide the basis of a principled approach for making decisions in complex contexts, such as the Italian legal system, where the current constitutional separation of powers can lead to an inadequate and incorrect assessment of risks and therefore to ineffective emergency management by the different levels of government.

In any case, scientific evidence is an essential prerequisite for better regulation by acting on the precautionary principle. To be cost effective, governments should take precautionary administrative measures based on scientific knowledge and thus carefully assess the risks they intend to manage.

Taking the potential and limits of the precautionary principle from the perspective we have outlined above into account might have an impact on governments’ ability to deal effectively with pandemic emergencies.

This matters in the case of Italy, where the current structure of the constitutional separation of powers between the Government, Regions and autonomous local authorities plays a crucial role in effectively managing the pandemic emergency.

### 3. Rules governing powers and competences in the constitutional scenario

Analysing the Italian constitutional scenario can provide substantial guidance for understanding the legal structure of powers and competences of Government, Regions and local authorities and explain why assessing pandemic risk can be impacted by a given separation of powers. Such an analysis can shed light on the administrative strategy implemented by the Government in the pandemic and enable us to evaluate its effectiveness in managing COVID-19 across the country.

First of all, we should bear in mind that the Italian Constitution (from now on the Constitution) does not explicitly refer to emergency power, except for a state of war (Article 78). However, this power has traditionally been included in the typical powers that the Constitution assigns to the Government.

In the constitutional system, the main rules governing the Government’s powers are established by Articles 76 and 77. Indeed, Parliament does not have a monopoly on legislative power, and the Government may also issue laws by two legal instruments that should be understood as extraordinary: legislative decree and decree-law.

In particular, Article 76 allows Parliament to delegate its legislative power to the Government, which in turn is given the power to issue legislative decrees. Hence, the legislative decree is a form of delegated law-making power, where Parliament may pass an enabling act entrusting the Government to adopt one or more acts that have legal force. Generally, the legislative decree is a legislative tool that is often deployed in all matters where a strong technical content is present.

The second extraordinary instrument, the decree-law, is provided for by Article 77. This is a form of law-making through emergency powers that the Government may exercise in “exceptional cases of necessity and urgency” and under “its own responsibility”.^59^ The Government can thus issue – without an enabling act from Parliament as required by the provisions of Article 76 – administrative measures that have the force of ordinary laws. However, such administrative measures will lose their effects as of the date of issue if Parliament does not transpose them into an ordinary law within sixty days of their publication.^60^

With the major reform on “administrative federalism” enacted by Law No. 3 of 18 October 2001, which amended Title V of the Constitution, Italy rapidly devolved legislative and regulatory powers to the Regions.^61^ Fundamentally, the constitutional amendment provided a new framework for the distribution of powers and competences between the national and local levels.^62^ It established a new institutional structure by dividing legislative and administrative competences and powers across the different levels of government.^63^

The amended articles of the Constitution are the basis for the fundamental reform of administrative federalism.

Article 114 recognises local authorities (Municipalities, Provinces, Metropolitan Cities) and Regions as autonomous entities of the State with their own statutes, powers and functions in accordance with the principles laid down in the Constitution.

Article 117 establishes the role and legislative powers of the State and Regions, indicating those matters for which the State has exclusive legislative power and those for which concurrent legislation of both the State and the Regions is possible. The Regions have exclusive power in all matters not expressly covered by State law. Municipalities, Provinces and Metropolitan Cities also have regulatory powers for the organisation and implementation of the functions attributed to them. Specifically, Article 117(3) establishes that the State and Regions have concurrent power, and the Regions have regulatory powers, in matters of public health.^64^ In this connection, at the national level, Parliament and Government are called upon to: (1) adopt fundamental health principles by means of framework laws and guidelines; and (2) establish essential levels of healthcare. At the regional level, the Regions implement: (1) general legislative and administrative activity; (2) the organisation of health facilities and services; and (3) the provision of healthcare based on specific local needs.

Article 118 provides for the subsidiarity principle, according to which all functions are exerted by Municipalities, while the possibility remains to confer them to higher levels of government in order to guarantee the uniform implementation of spending functions across the country.

Article 120 guarantees national unity and the unitary nature of the constitutional system by providing for the government’s substitution power.^65^ According to Article 120(2), the Government can act for the Regions and other local authorities if: (1) the latter fail to comply with international rules and treaties or EU legislation; (2) in the case of grave danger for public safety and security; or (3) whenever such action is necessary to preserve legal or economic unity and in particular to guarantee the basic level of benefits relating to civil and social entitlements, regardless of the geographical borders of local authorities. To this end, the law shall lay down the procedures to ensure that (4) subsidiary powers (ie the government’s substitution power) are exercised in compliance with the principles of “subsidiarity” and “loyal cooperation”.

Lastly, with regards to powers and competences in emergencies, it should be noted that in the Italian legal system several authorities can introduce specific regulatory acts establishing administrative measures needed to deal with emergencies in accordance with the Constitution.

The power of ordinance has a particular role in managing emergencies, as it can be exercised in situations of necessity and urgency. In particular, the legal system provides for: (1) ordinances of the Minister of Health, the Presidents of Regions and Mayors in accordance with Article 32 of Law 833/1978 (National Health Service Act); (2) ordinances of the public security authority pursuant to Law 225/1992 (National Civil Protection Service Act); (3) local ordinances adopted by the Mayor in public health emergencies in accordance with Article 117 of Legislative Decree 112/1998; (4) ordinances of the Mayor in accordance with Article 54 of Law 267/2000 (Local Authorities Act); and (5) Article 1(2) of Legislative Decree 112/1998 allows for the adoption of “emergency administrative measures” by the Regions and Municipalities in all other cases permitted by law.^66^

As we will see, the structure of power just described highlights the problem of risk assessment among the institutional actors involved in the administrative decision-making process. Though the current system of allocation of powers and competences to the Regions and other local authorities might be an advantage in terms of correctly assessing and managing risk in their areas, at the national level, this system requires an effective sharing of powers and strategies between the centre and the periphery, where the measures of the Regions and local authorities must be adopted in accordance with the measures advanced by the Government, and vice versa.

Since correct risk assessment by an authority must take the characteristics of its area into account – data on the epidemiological situation, for example, or on the average age of the population, and the capacity of the health system with regards especially to the availability of intensive care beds – it might be assumed that in the Italian legal system’s effective risk assessment could be facilitated by the specific competences established by the constitution for the Regions and other local authorities in health matters.

However, as I will argue, this is a theoretical advantage that works only if power is effectively shared between the different levels of government.

In fact, in order to provide an adequate and correct risk assessment at the national level and take effective measures to contain and manage the pandemic, the current system needs powers and strategies to be shared between local authorities, Regions and the Government.

## IV. Why the administrative strategy is ineffective

### 1. Sharing emergency administrative powers

Sharing administrative powers at all levels of government is an important part of the task of States.^67^ Indeed, enhancing multi-level regulatory governance has become a priority in many EU States. For this reason, the EU supports sharing of administrative regulatory powers by encouraging better regulation at all levels of government, calling on the Member States to improve coordination and avoid overlapping responsibilities among regulatory authorities.^68^

In Italy, until the adoption of Constitutional Law 3/2001, regulatory reform had been promoted, designed and implemented mainly at the national level. With the reform, as we have seen (Section III.3), such a centralised approach lost legal and political ground. At the same time, responsibilities for developing and implementing administrative regulation policies have not been explicitly allocated to either the State, the Regions or the local authorities. Hence, the responsibility for administrative regulation and regulatory reform lies with each of the levels of government in the matters where they exert legislative powers. In like manner, there is no overall competence at the central level to monitor and control regulatory reform programmes at the local level. Accordingly, the new constitutional structure calls for effective sharing of administrative powers across the different levels of government.

On the basis of the analysis carried out so far, I will now argue that the main problems of the Italian administrative strategy for the COVID-19 pandemic are due to the lack of effective sharing of administrative powers and, more specifically, to the failure to share regulatory powers across the different levels of government with the participation and cooperation of all institutional actors involved in the emergency decision-making process: the Government, Regions and local authorities. In particular, this problem has impacted the risk assessment of the various authorities called upon to manage the health emergency. As a result, the problem has impacted nationwide risk assessment and, consequently, the management of the emergency at the national level, leading to the adoption of inconsistent measures by the various institutional actors involved in the administrative decision-making process.

In particular, I discuss this problem in Italian policies from two key points of view: the Government’s administrative strategy for managing the virus’s spread by means of the “incremental approach” (Section IV.1.a) and the Government’s administrative strategy for implementing the nationwide pandemic health plan (Section IV.1.b). In doing so, I shall take into account the considerations presented above concerning emergency risk regulation (Section III.1), the precautionary principle (Section III.2) and the rules governing powers in the constitutional scenario (Section III.3).

#### a. Rethinking the government’s incremental approach

One of Italy’s main problems in relation to the ineffective sharing of administrative powers for managing the pandemic is clearly displayed in what I will call the “incremental approach”.^69^

This approach is essentially based on the “progressive” application of emergency measures by the Government in order to manage the “exponential” spread of the virus. The Italian administrative strategy for the pandemic is fundamentally founded on such an approach. In fact, as we have seen (Section II), the Government addressed the pandemic by enacting several decrees (DPCMs) that “progressively increased” restrictions in lockdown areas (red zones), which were then extended from time to time until they finally applied to the entire country in the national lockdown.

In my opinion, although the incremental approach may be a correct application of the principle of proportionality, given the Government’s proportionate use of emergency powers in dealing with the pandemic, it is the result of an ineffective sharing of administrative regulatory powers between the Government, Regions and local authorities. Indeed, the progressive enforcement of lockdown areas, which from time to time increased the extent and severity of the emergency measures, demonstrates the difficulty of governing the spread of the virus in the red zones rather than the effective implementation of a proportionate administrative strategy. And this is mainly due to the lack of effective cooperation between the Government and the Regions in exercising their respective emergency powers.

From a general point of view, the incremental approach reveals the limited effectiveness of the national and local measures and strategies for managing and containing the pandemic when those measures and strategies are not shared.

I argue that even the stringent national lockdown^70^ is essentially the result of the ineffective sharing and planning of administrative measures and strategies for managing the pandemic across the different levels of government and especially, in this case, between the Government and the Regions. One can legitimately wonder whether the Government can adopt an effective administrative strategy for managing the emergency without sharing and planning their measures with those of the Regions.

From this perspective, we can say that the Government’s incremental approach has proven ineffective in coping with the pandemic. I will now explain why in the following points.

(1) Regarding risk assessment for pandemics, the science^71^ shows that the spread of COVID-19 is rapid and exponential. Consequently, the incremental approach does not work if it is not properly implemented with the effective participation of all institutional actors involved in managing the pandemic. Scientific data^72^ and statistics^73^ on the spread of the virus were not predictive of what the situation would have been in the short and medium term. Hence, a correct risk assessment of the virus’s nationwide spread would have suggested that the administrative measures and, more generally, the strategies should have been shared among all players involved in the main strategy. Very often, however, the Government’s strategy has not been in line with those of the Regions, revealing an inadequate assessment of the risk that the virus would spread throughout the country, and thus the ineffective sharing of emergency powers. In fact, some important emergency measures implemented by the Regions clearly contradict the Government’s main strategy.

To take a few examples,^74^ Marche Region Ordinance No. 1 of 25 February 2020, issued pursuant to Decree-Law No. 6 of 2020, established measures that were more stringent than the Government’s, disregarding the latter’s strategy. For this reason, the Government contested the order before the court.^75^ Although a judgment in favour of the Government was handed down and the challenged ordinance was suspended, the Marche Region legitimately adopted a new ordinance establishing emergency measures based on the same Decree-Law No. 6/2020, once again disregarding the Government’s strategy. Another paradigmatic case is provided by a series of ordinances by the Campania Region aimed at imposing a more stringent lockdown at the local level than the lockdown established by the Government at the national level. Unlike the Marche case, the ordinances of the Campania Region, although contested before the administrative judge, were not suspended, thus making the Government’s strategy ineffective.^76^

Consequently, in the absence of effective sharing and planning of the main strategy with the Regions, the Government had to ‘increase’ the emergency measures from time to time until finally imposing the stringent national lockdown.

(2) In the absence of power sharing and strategies based on correct risk assessment at the national level, the Government’s incremental approach seems to have played a considerable role in people’s behaviour, inducing them to make “bad choices”. As the data show,^77^ the Government’s incremental lockdown of Municipalities, Provinces and Regions in northern Italy induced masses of people to move towards the southern regions, spreading the virus to parts of Italy that had not yet been affected. An emblematic case of this kind took place immediately after the DPCM of 8 March 2020 (see Section II) locked down Lombardy and another fourteen provinces in northern Italy, spurring thousands of people to flee to the south.

Such potential negative externalities, as well as other negative spill-overs or distortions, should have suggested that the Government share its regulatory acts with those of the “target” Regions (ie the northern Regions), as well as with the other Regions that could be indirectly jeopardised by the lockdown measures (ie the southern Regions). Alternatively, the Government should have undertaken to coordinate the strategies of the regions and local authorities in order to enhance the adoption of effective control measures for people exiting the red zones and entering less affected regions.^78^

More generally, in applying lockdown measures, the Government should have shared and planned its strategy with the Regions on the basis of a common risk assessment that took into account not only the regional territories, but the entire country. Accordingly, the Government should have established effective countermeasures together with all of the Regions potentially involved in lockdown decisions to prevent the virus from spreading from high-risk to low-risk areas. An effective emergency response must be coordinated as a consistent system of actions taken simultaneously by the different actors involved in the decision-making process.

(3) The Government’s incremental approach also revealed the problem of effectively sharing and planning precautionary measures (see Section III.2) across the different levels of government. The critical situation that arose because of the epidemic’s severity called for effective testing of symptomatic and asymptomatic cases, as well as proactive tracing of potential positives across the country. On this point, these precautionary measures were supported by scientific data on the transmission of COVID-19 by asymptomatic people.^79^

The absence of a shared strategy for the adoption and implementation of precautionary measures proved particularly harmful in regions where the epidemic risk is higher. Indeed, it is no coincidence that the outbreak spread so quickly in northern Italy and especially in Lombardy. In this Region, the efficient public rail transport network connecting urban areas, large numbers of commuters^80^ and high levels of air pollution^81^ are thought to have increased the incidence of infection. From this point of view, it is clear that risk assessment has been inadequate, and strategies have thus been ineffectively shared between Lombardy and the Government.

The Government should have promoted an effective precautionary strategy for health checks by sharing it with the strategies of the Regions and ensuring efficient nationwide implementation on the basis of a global risk assessment. Conversely, data on infections and deaths reveal that strategies were not shared effectively with the hardest-hit Regions.

(4) The incremental approach shows that most of the problems of administrative strategy are also motivated by political issues between parties governing regions and belonging to the coalition now governing the country.

From the time when the virus began to spread, the multi-level management of the emergency has triggered competition and institutional division between the Government and Regions^82^ due to policymakers’ political differences. The management of the pandemic, in fact, has thrown light on the deep political division between the Government, led by the coalition of left-wing parties such as the Democratic Party and the Five Star Movement, and the hardest-hit Regions – Lombardy and Veneto – led by traditionally right-wing populist parties such as the League and Brothers of Italy.

In particular, many of the administrative measures taken by the Regions were in contrast with the Government’s strategy, largely for political reasons. From this standpoint, it can be seen that there has been an “institutional clash” between the regional governments and the national government on the political and administrative actions to be taken to effectively manage the emergency. It is no coincidence that the Government’s Minister of Health is a member of one of the opposition parties in Lombardy and Veneto, and that the Governors of Lombardy and Veneto belong to the coalition opposing the Government.

To give a few specific examples, a bitter dispute has occurred between Prime Minister Giuseppe Conte and Attilio Fontana, Governor of Lombardy and member of the right-wing populist party League, with regards to the ineffective management of the emergency in the region most affected by the virus. Similarly, as we have seen, Luca Ceriscioli, Governor of the Marche Region and member of the centre-left party in the majority coalition, opposed the Government’s decision to declare a state of emergency only in the northern Regions.^83^

In essence, these strong political divisions have impacted effective power sharing among the different levels of government, causing problems for the Government’s incremental administrative strategy.

(5) The incremental approach also shows the important role that scientific competence plays in emergency management.^84^

In this regard, one of the main goals of scientific expertise is to inform and legitimise governments’ decisions, especially in high-uncertainty situations relating to public health. During the COVID-19 outbreak, scientific and technical experts have assisted central and regional governments by contributing to the content of decisions and, more generally, of administrative emergency management strategies. As scientific evidence is the basis for sound political choices, scientific and technical experts have become part of the rationale of governments’ decisions and have been useful in reassuring the public with concrete solutions.^85^

Indeed, in the immediacy of a pandemic, as is logical to assume, the demand for scientific expertise increases as governments search for certainty in understanding problems and choosing effective measures for managing the emergency. Especially in the most delicate phases of an emergency, scientific expertise is useful in informing, legitimising and justifying government evaluations and responses to problems, even as political and administrative considerations continue to govern such choices. The result is an increased reliance on scientific expertise and politicisation of scientific and technical information.^86^

By invoking scientific expertise, policymakers create the need for what is perceived as evidence-based policymaking, which suggests to the public that political and administrative decisions are based on reasoned and informed judgments^87^ aimed at ensuring the public interest and guaranteeing individual rights.

However, a major problem is that scientific expertise might obscure the accountability of decisions. As scientific and technical experts serve to inform and legitimise political and administrative decisions, they may also obscure responsibility for policy responses and outcomes.^88^ Scientific expertise helps to establish the severity of a pandemic in a population, to understand the epidemiological trend over time and to evaluate the effects of political and administrative measures, from mitigation to suppression. Nonetheless, undertaking policy actions is the responsibility of government leaders. As scientific expertise becomes more prominent in the policy process, who is accountable for policymaking becomes more obscure.^89^

To work better in emergencies, scientific expertise also requires effective sharing of administrative powers based on accurate risk assessment, as I will now explain.

In Italy, since the beginning of the virus’s spread, the various institutional actors, especially the Government and the Regions, have established their own scientific task forces to support administrative measures and strategies in managing the pandemic.

The main problem is that, by doing so, risk assessment at the national level is fragmented. Conflicts can also arise between institutional actors involved in the decision-making process. In this scenario, indeed, the Government and the Regions have adopted administrative decisions and strategies based on the risk assessments provided by their own central and regional task forces. It should be noted that this situation, like others discussed here, derives from the current constitutional architecture of separation of powers where the decision-making process is assigned to the different levels of government.

However, managing a pandemic requires a comprehensive risk assessment. Italian policies matter, as they show how, at the beginning of the pandemic, some Regions’ task forces underestimated COVID-19, while other Regions gave it a certain importance. This behaviour on the part of policymakers was not led by the Government, which, on the contrary, criticised the regional governments’ solutions. The outcome, as I claimed for the incremental approach, is that the Government’s measures and strategies are not shared with those of the Regions and vice versa, and policymakers’ accountability is obscured by invoking scientific expertise for pandemic management decisions.

#### b. Implementing the national pandemic health plan

There is no doubt that a pandemic affects the whole of society. No single organisation can effectively prepare for a pandemic in isolation, and uncoordinated preparedness of interdependent public organizations will reduce the ability of the health sector to respond.^90^ A comprehensive, shared, coordinated, whole-of-government approach to pandemic preparedness is required.^91^

The Government’s strategy, as we have seen in the incremental approach to dealing with the emergency, proved particularly ineffective due to the failure to share administrative powers with the other institutional actors involved in the pandemic decision-making process, particularly the Regions. But this, as we shall see now, was not the only weak point.

I will argue here that another of the major problems was the lack of effective implementation of the national pandemic health plan. In particular, we will see how and why the ineffective implementation of the plan by the Government, Regions and local authorities posed serious problems for containing the spread of the virus and, more specifically, for avoiding the collapse of the public healthcare system.

On this point, one of the main problems for public health posed by the novel coronavirus is its ability to spread with exceptional ease and speed,^92^ threatening to overwhelm the healthcare system. In particular, what should be especially clear from the data is the critical situation of the intensive care system in Italy,^93^ which has been severely weakened by the pandemic.^94^ Consequently, to mitigate the risk of the healthcare system’s collapse, the Government should verify the actual capacity of the intensive care system at the national level, cooperating with the Regions and local authorities to ensure that critical care bed availability is efficiently managed.

In this case, effective actions shared among all institutional actors and based on an adequate and accurate risk assessment at the national level would avoid saturating the intensive care system in the medium and long term, while the Government should be able to increase capacity in the short term. Yet, the data on the intensive care system show that the situation was inefficiently managed in the Regions hardest hit by COVID-19, especially in Lombardy, which paid a high price at the local level for the ineffective implementation of the pandemic health plan at the national level.

More generally, it should be emphasised that this point also demonstrates the importance of sharing administrative powers between Government, Regions and local authorities to implement the pandemic management plan effectively throughout the country. In this connection, many elements based on scientific and epidemiological data demonstrate that the COVID-19 pandemic called for effective cooperation and coordination across all levels of government. In addition, it must be borne in mind that fighting a pandemic hinges on many factors, most of which are time consuming or in any case cannot be accomplished quickly. Preparing a candidate vaccine, for example, takes a long time in terms of both preclinical and clinical development. Likewise, developing and testing an effective drug involves complex multi-stage clinical trials.

Such considerations might be sufficient on their own to justify taking effective actions to mitigate the pandemic emergency’s impact on the public healthcare system. In this phase, as we have seen, emergency risk regulation requires that regulatory action be taken in the immediacy of an emergency in order to mitigate its impact (Section III.1). To avoid the collapse of the public health system, the Government should thus have contained the spread of the virus by effectively implementing the nationwide pandemic management plan with the participation of all institutional actors.

The WHO has recognised the importance of sharing administrative powers through the participation and cooperation of the various institutional actors involved in the strategy against pandemics. In this regard, the WHO has drawn up specific Guidelines^95^ for implementing a pandemic influenza preparedness plan^96^ that States should apply in order to manage the spread of the virus throughout their territories. In particular, the WHO’s Guidelines encourage States to develop efficient plans, based on national risk assessments, with the effective participation of institutional actors at all levels of government.

In Italy, the most serious problem is that the Government, although it had already developed its own national plan,^97^ neither implemented it effectively^98^ nor tried to foster its effective adoption by the Regions and local authorities, disregarding a crucial point of the WHO’s Guidelines. Consequently, the failure to implement the national pandemic plan, as we have seen, created the conditions for the collapse of the public health system, with the overcrowding of intensive care units and the consequent loss of life.

***

In conclusion, the Italian policies regarding the COVID-19 outbreak can demonstrate the importance of: (1) rethinking the incremental approach; and (2) implementing a national health plan for pandemics by sharing powers, and more specifically administrative regulatory powers for emergencies based on an adequate and accurate risk assessment at the national level, among the different levels of government with the participation, cooperation and coordination of all institutional actors involved in the pandemic decision-making process.

### 2. Possible solutions from the constitutional perspective

As we have seen, sharing administrative powers at the different levels of government plays a particularly important role in managing emergencies in the constitutional scenario, where competences are distributed between Government, Regions and local authorities, and several institutional actors are allowed to adopt regulatory acts (see Section III.3).

The major changes that the constitutional amendments have brought to policymaking in the Italian legal system require that constant support be provided to the Regions and local authorities, especially in emergencies. Despite significant decentralisation, the Government still has a fundamental role to play in sharing and coordinating administrative powers at the different levels of government and in ensuring loyal cooperation among all of the institutional actors involved in emergency decision-making processes.

Indeed, the Government is tasked with promoting and coordinating “action with the Regions” (Article 5 of Law 400/1988), as well as with advancing cooperation “between the State, Regions and local authorities” (Article 4 of Legislative Decree 303/1999).^99^ Similarly, the Government must promote “the necessary actions for the development of relations between the State, Regions and local authorities” and ensure the “consistent and coordinated exercise of the powers and remedies provided for cases of inaction and negligence” (Article 4 of Legislative Decree 303/1999).

Looking at the constitutional perspective, some possible solutions might be proposed.

(1) In the Italian constitutional scenario, although concurrent power to legislate on matters of public health is vested in the State (ie the Government) and the Regions pursuant to Article 117(3), the State (ie the Government and the Regions together), on the basis of the principle established by Article 32(1), “safeguards health as a fundamental right of the individual and as a collective interest”.

I argue, more specifically, that safeguarding health is a task of the State based on the fundamental principle of the Constitution referred to in Article 3(2), where the duty of the State is to “remove those obstacles of an economic or social nature” that, by constraining the “freedom and equality of citizens”, impede the “full development of the human person and the effective participation of all workers in the political, economic, and social organisation of the country”.

Thus, I believe that under the joint interpretation of Article 3(2) and Article 32 of the Constitution, as well as the principle of loyal cooperation, the Government and the Regions must act by sharing administrative powers (and strategies) among them in order to protect the fundamental right to health.

In so doing, the Government can play an essential role in promoting institutional balance and cooperation between the national and local levels, maximising loyal cooperation and implementing vertical and horizontal subsidiarity.

(2) Sharing administrative powers for emergencies can also be encouraged and enhanced through the effective implementation of constitutional tools, such as the system of Conferences based on the principle of loyal cooperation.

(a) The Conference on the Relationships between Government, the Regions and the self-governing Provinces is the key legal tool for multi-level political negotiation and collaboration. It serves in an advisory, normative and planning capacity and acts as a platform facilitating power sharing.

(b) The Conference on the Relationships between Government and the Municipalities coordinates relations between the Government and local authorities through studies, information and discussion of issues affecting local authorities.

(c) The Permanent Conference on the Relationships between Government, the Regions and the Municipalities deals with areas of shared competence.^100^

(3) In order to “safeguard health as a fundamental right of the individual and as a collective interest”, Article 120(2) of the Constitution could be applied whenever it is necessary to guarantee “the national unity and the unitary nature of the constitutional system”.

I claim that this provision, which establishes the Government’s administrative substitution power, provides for the centralisation of administrative powers in specific cases contemplated by the Constitution. In this sense, Article 120(2) lays down that the Government can act for the Regions and/or local authorities in cases of “grave danger for public safety and security”. In the light of this definition, the Government’s substitution for the Regions and/or local authorities might be invoked as a result of the “grave danger for public safety”, as well as in order to preserve “economic unity” and guarantee the “basic level of benefits relating to civil and social entitlements”.

In my view, however, the Government should exercise its power of substitution as an *extrema ratio* whenever effective sharing among all of the institutional actors has not been implemented. Article 120(2) is clear in this regard, requiring that the substitution power be exercised in compliance with the principles of “subsidiarity” and “loyal cooperation”.

In particular, I maintain that this power would have been useful as a remedy for inaction or delay on the part of the Regions and local authorities in implementing the national pandemic health plan or adopting emergency measures. In such cases, substitution powers may be granted to the central level of government (the appropriate ministry or the Council of Ministers), which intervenes instead of the local level (the Regions and/or local authorities).

The same considerations on the Government’s administrative substitution power can be applied to the following solutions as “*ex post* remedies” for the failure to share administrative powers effectively across the different levels of government.

(4) The Government has extraordinary administrative power to annul illegitimate measures (eg ordinances) issued by local authorities in order to “safeguard the unity of the national legal system” (Article 138 of Law 267/2000^101^ and Article 2 of Law 400/1988).^102^ The power, generally applied to measures issued by the Municipalities, can be extended to regional measures on the basis of Article 120(2) of the Constitution.^103^

(5) The Government has the right of action to challenge illegitimate measures issued by the Regions and other local authorities before the court, in accordance with the constitutional provisions governing judicial review in the Italian administrative justice system (Articles 24, 101, 103, 111, 113 and 125 of the Constitution).

## V. Conclusion

In this article, starting from the main regulatory acts and considering scientific knowledge and epidemiological data on COVID-19, I have analysed the administrative measures and strategies for fighting the pandemic implemented by the Government in the immediacy of the emergency.

Specifically, I carried out this analysis to verify this strategy’s effectiveness at managing the pandemic throughout the country.

In analysing the administrative strategy, I emphasised the role that Italy’s current system of constitutional separation of powers plays in emergency management and how this system can impact health risk assessment.

To do so, I first illustrated the risk management system in Italian and EU law and discussed some key legal issues that are useful for clarifying the main points of the matter and for understanding the weaknesses of the administrative strategy with a view to proposing solutions that the Government might take into account in adopting its strategy.

In particular, I briefly examined the notion and features of emergency risk regulation (Section III.1), the potential and limits of the precautionary principle (Section III.2) and the rules governing powers and competences in the Italian constitutional scenario (Section III.3).

To summarise the main points of this analysis, I stressed that the Italian policies for managing the pandemic demonstrate the importance of effective “sharing of administrative powers” – and more specifically the administrative regulatory powers for emergencies – based on an adequate and accurate risk assessment, across the different levels of government with the participation, cooperation and coordination of all institutional actors involved in the emergency decision-making process: the Government, Regions and local authorities.

Fundamentally, I emphasised that the Italian case reveals the importance of sharing administrative powers from two main points of view.

First, I argued that the “incremental approach” to dealing with the emergency, although based on the proportionate use of powers, is largely ineffective or even harmful in the absence of cooperation among all actors – the Regions and local authorities – involved in the main strategy implemented by the Government (Section IV.1.a).

Second, I discussed the importance of cooperation between the Government, Regions and local authorities for the effective and efficient implementation of a nationwide pandemic health plan (Section IV.1.b).

I suggested that these points be viewed from a constitutional perspective in order to propose some possible solutions. From this perspective, the problems of effective sharing of administrative powers across the different levels of government could be resolved by systematically interpreting the Constitution and implementing specific constitutional tools provided by the legal system (Section IV.2).

In conclusion, more generally, I argue that – and this is the main thrust of the article – administrative powers should be shared across the different levels of government based on an adequate and accurate risk assessment with the participation and cooperation of all of the institutional actors involved in the emergency decision-making process in order to safeguard the fundamental rights enshrined in the Constitution as well as in EU and international law.

In pandemics, this aim must be achieved not only to guarantee the right to health, but also to safeguard all of the rights that might be jeopardised by the exercise of administrative powers and, more specifically, the exercise of emergency powers in dealing with the pandemic. The strong measure of “lockdown”, for example, should be the *extrema ratio* of administrative powers because it suspends the rule of law and jeopardises rights. Indeed, as I have claimed in analysing the Italian policies, sharing powers with effective cooperation between Government, Regions and local authorities in managing the pandemic would optimise the adoption of nationwide virus containment measures, avoiding or at least delaying the application of stringent emergency measures such as the lockdown of Municipalities, Provinces, Regions or even the entire country.

Taking into consideration the correct application of emergency risk regulation (Section III.1) and the precautionary principle (Section III.2), although lockdowns aim to contain specific areas that are most affected by the virus, they must be proportional to the risk that they intend to curtail. When such measures are adopted to protect the right to health, as is the case in a pandemic, this right must be balanced with other rights. Yet, if administrative powers are not shared effectively across the different levels of government, the balancing principle might be disregarded by jeopardising one or more rights without legitimate justification (eg the right to freedom of movement enshrined in Article 16 of the Constitution).

This is the problem that the Italian policies bring to light: a problem that I believe that the Government must take into account in the near future as it strives to manage COVID-19 and other similar pandemics.

## Footnotes

1 COVID-19 is the name of the disease caused by the novel coronavirus SARS-CoV-2.

2 WHO, “Statement on the second meeting of the International Health Regulations (2005) Emergency Committee regarding the outbreak of novel coronavirus (2019-nCoV)”, Geneva, Switzerland, 30 January 2020 <https://www.who.int/news-room/detail/30-01-2020-statement-on-the-second-meeting-of-the-international-health-regulations-(2005)-emergency-committee-regarding-the-outbreak-of-novel-coronavirus-(2019-ncov)>. PHEIC has been defined in the International Health Regulations (IHR) of 2005 as an extraordinary event which can: (1) constitute a public health risk to other states through the international spread of disease; and (2) potentially require a coordinated international response. Furthermore, this definition implies a situation that is: (1) serious, unusual or unexpected; (2) carries implications for public health beyond the affected State’s national borders; and (3) and may require immediate international action.

3 WHO, “Director-General’s opening remarks at the media briefing on COVID-19”, 11 March 2020 <https://www.who.int/dg/speeches/detail/who-director-general-s-opening-remarks-at-the-media-briefing-on-covid-19---11-march-2020>.

4 Resolution of the Council of Ministers of 31 January 2020, adopted pursuant to Legislative Decree 1/2018 (Civil Protection Code) <https://www.gazzettaufficiale.it/eli/id/2020/02/01/20A00737/sg>. On the declaration of emergency rule, see European Commission for Democracy through Law (Venice Commission) <https://www.venice.coe.int/webforms/documents/default.aspx?pdffile=CDL-STD(1995)012-e>.

5 DPCM of 9 March 2020 <www.gazzettaufficiale.it/eli/gu/2020/03/09/62/sg/pdf>.

6 For the general framework of all measures adopted by the Italian State during the COVID-19 emergency, see <https://www.camera.it/temiap/documentazione/temi/pdf/1203754.pdf?_1588279335853>.

7 FG Nicola, “Exporting the Italian Model to Fight COVID-19” (The Regulatory Review, 23 April 2020) <https://www.theregreview.org/2020/04/23/nicola-exporting-italian-model-fight-covid-19/>.

8 Decree-Law 6/2020 <https://www.gazzettaufficiale.it/eli/id/2020/02/23/20G00020/sg>, converted, with amendments, by Law No. 13 of 5 March 2020 <https://www.gazzettaufficiale.it/eli/id/2020/03/09/20G00028/sg>.

9 Decree-Law 6/2020 also allowed the Presidents of the Regions to adopt “further emergency measures in cases of extreme urgency”.

10 It should be noted that the Government’s administrative regulatory acts (the DPCMs) do not violate the statutory reservation, inasmuch as they are based on Decree-Law 6/2020. Nevertheless, there are some aspects that call the legality of the Government’s regulatory acts into question. For the recent debate on this point, see M Simoncini, “The Need for Clear Competences in Times of Crisis. Clashes in the Coordination of Emergency Powers in Italy” (Verfassungsblog, 9 April 2020) <https://verfassungsblog.de/the-need-for-clear-competences-in-times-of-crisis/>; A Vedaschi and C Graziani, “Coronavirus Emergency and Public Law Issues: an Update on the Italian Situation” (Verfassungsblog, 12 March 2020) <https://verfassungsblog.de/coronavirus-emergency-and-public-law-issues-an-update-on-the-italian-situation/>; D Tega and M Massa, “Fighting COVID 19 – Legal Powers and Risks: Italy” (Verfassungsblog, 23 March 2020) <https://verfassungsblog.de/fighting-covid-19-legal-powers-and-risks-italy/>.

11 DPCM of 8 March 2020 <www.gazzettaufficiale.it/eli/gu/2020/03/08/59/sg/pdf>.

12 DPCM of 9 March 2020 <www.gazzettaufficiale.it/eli/gu/2020/03/09/62/sg/pdf>.

13 DPCM of 11 March 2020 <www.gazzettaufficiale.it/eli/gu/2020/03/11/64/sg/pdf>.

14 In the Italian legal system, the administrative powers of the Government (including DPCMs) and other national administrative authorities (particularly of Ministers) are specifically regulated by Law No. 400 of 23 August 1988 (Arts 14–17) <https://www.gazzettaufficiale.it/eli/id/1988/09/12/088G0458/sg>.

15 Further measures implementing Decree-Law 6/2020 are provided by the DPCM of 22 March 2020 <https://www.gazzettaufficiale.it/eli/gu/2020/03/22/76/sg/pdf>.

16 Order of 20 March 2020 <https://www.gazzettaufficiale.it/eli/id/2020/03/20/20A01797/sg>.

17 Order of 28 March 2020 <https://www.gazzettaufficiale.it/eli/id/2020/03/29/20A01921/sg>.

18 Decree-Law 19/2020 <https://www.gazzettaufficiale.it/eli/id/2020/03/25/20G00035/sg>.

19 For an analysis of various ordinances enacted by the Regions in order to manage the COVID-19 pandemic, see Simoncini, supra, note 10; see also V Di Capua, “Il nemico invisibile. La battaglia contro il Covid-19 divisa tra Stato e Regioni” [The Invisible Enemy: The Battle against Covid-19 between State and Regions] (2020) 1 Federalismi 1–28; For an analysis of municipal ordinances, see, eg, I Forgione, “La gestione locale dell’emergenza da Covid-19” [Local Emergency Management for Covid-19] (2020) 2 Diritto dell’Economia 92–100.

20 In particular, Art 2(3) of Decree-Law 19/2020 did not affect the effects produced and acts adopted on the basis of decrees and ordinances issued pursuant to Decree-Law 6/2020 or Art 32 of Law 833/1978, and established that the measures previously adopted by the DPCMs of 8 March 2020, 9 March 2020, 11 March 2020 and 22 March 2020 as still in force on the date of entry into force of the said Decree-Law shall continue to apply within the original terms.

21 See A Dicey, *Introduction to the Study of Constitutional Law* (Carmel, IN, Liberty Fund 1982). For a more recent discussion of the topic in EU law, see O Beaud, “The Allocation of Competences in a Federation. A General Introduction” in L Azoulai (ed.), *The Question of Competences in the European Union* (Oxford, Oxford University Press 2014) pp 19–23 and 34–35.

22 See LR Perfetti, “I diritti sociali. Sui diritti fondamentali come esercizio della sovranità popolare nel rapporto con l’autorità” [Social Rights. On Fundamental Rights as the Exercise of Sovereignty in the Relationship with Authority] (2013) 1 Diritto pubblico 61.

23 P Norris, *Driving Democracy: Do Power Sharing Institutions Work?* (Cambridge, Cambridge University Press 2008).

24
A Alemanno (ed.), Governing Disasters: The Challenges of Emergency Risk Regulation (Cheltenham, Edward Elgar 2011) p XIX.

25 However, the notion of risk in Italian administrative law is analysed by M Simoncini, *La regolazione del rischio e il sistema degli standard. Elementi per una teoria dell’azione amministrativa attraverso i casi del terrorismo e dell’ambiente* [*Risk Regulation and the Standards System. Elements for a Theory of Administrative Action through the Cases of Terrorism and the Environment*] (Napoli, Editoriale Scientifica 2010) chs 2 and 4, where the author postulating the notion of risk argues and suggests, in an innovative approach, the transition from the “emergency” perspective to the “risk regulation” perspective.

26 For this contention, see the seminal work of MS Giannini, “Potere di ordinanza e atti necessitati” [Power of Ordinance and Compelled Acts] (1948) Giur Compl Cass Civ 951–53; see also MS Giannini, *Lezioni di diritto amministrativo* [*Lessons in Administrative Law*] (Milan, Giuffrè 1950) p 102; MS Giannini, *Diritto amministrativo* [*Administrative Law*] (Milan, Giuffrè 1993) p 267. On the argument, see also V Angiolini, *Necessità ed emergenza nel diritto pubblico* [*Necessity and Emergency in Public Law*] (Milan, Giuffrè 1986) p 143.

27 M Simoncini, “Regulating Catastrophic Risks by Standards” (2011) 1(2) European Journal of Risk Regulation 39, where the author explains the public law approach to “emergency” and “risk regulation”.

28
ibid, 91.

29 U Beck, “Risk Society Revisited” in B Adam, U Beck and J van Loon (eds.), *The Risk Society and Beyond: Critical Issue for Social Theory* (London, Sage 2000) pp 211–29; see also the seminal work by U Beck, *Risikogesellschaft. Auf dem Weg in eine andere Moderne* (Frankfurt am Main, Suhrkamp Verlag 1986). Beck is responsible for analysing the sociopolitical dimension of risk management and in particular the problem of the relationship between science and society through the criticism of the monopoly that scientific rationality currently holds.

30 Alemanno, supra, note 24, XXII.

31
ibid, XXII–XXIII. See also DD Caron, “Addressing Catastrophes: Conflicting Images of Solidarity and Self Interest” in DD Caron and CH Leben (eds), *Les aspects internationaux des catastrophes naturelles et industrielles* [*The International Aspects of Natural and Industrial Catastrophes*] (Hague, The Hague Academy of International Law 2001); ML Dauber, “Fate, Responsibility and ‘Natural’ Disaster Relief: Narrating the American Welfare State” (1999) 33 Law & Society Review 257–61.

32 U Rosenthal, RA Boin and LK Comfort (eds), *Managing Crises: Threats, Dilemmas, Opportunities* (Springfield, IL, Charles C. Thomas 2001).

33 Alemanno, supra, note 24, XXII.

34
ibid, XXIII. According to the author, “suddenness” is the most relevant criterion for defining a pandemic for the purposes of emergency regulation.

35 N Madhav et al, “Pandemics: Risks, Impacts, and Mitigation” in DT Jamison et al (eds), *Disease Control Priorities: Improving Health and Reducing Poverty* (Washington, DC, The International Bank for Reconstruction and Development/The World Bank 2017).

36 MB Gerrard, “Emergency Exemptions from Environmental Laws after Disasters” (2006) 20(10) Natural Resources & Environment 10–14.

37 G Martinico and M Simoncini, “Emergency and Risk in Comparative Public Law” (Verfassungsblog, 9 May 2020) <https://verfassungsblog.de/emergency-and-risk-in-comparative-public-law/>. According the authors, it is the facts and not the law that indicate the conclusion of an emergency. Thus, the risks posed by the use of extraordinary administrative measures should be considered, especially at the end of the emergency when the government’s powers should be subject to legal control in order to avoid departures from original objectives. In the same sense, see also Simoncini, supra, note 27, 39.

38 On the state of exception, see C Schmitt, *Die Diktatur: Von den Anfängen des modernen Souveränitätsgedankens bis zum proletarischen Klassenkampf* (Berlin, Duncker & Humblot 1989). Schmitt’s jurisprudential thinking placed the state of exception at the very centre of analysis, beginning with his work on the Roman dictatorship.

39 Martinico and Simoncini, supra, note 37.

40 See, eg, R Harding and E Fisher (eds), *Perspectives on the Precautionary Principle* (Alexandria, Federation Press 1999); N De Sadeleer, “Les avatars du principe de precaution en droit public (effet de mode au revolution silencieuse)?” (2001) 17 Revue Française de Droit Administratif 547; A Alemanno, “Le principe de précaution en droit Communautaire: stratégie de gestion des risques ou risque d’atteinte au marché intérieur?” (2001) 4 Revue du droit de l’Union européeenne 917; E Fisher, “Precaution, Precaution Everywhere: Developing a ‘Common Understanding’ of the Precautionary Principle in the European Community” (2002) 9 Maastricht Journal of European and Comparative Law 7. For a general discussion, see P Craig, *EU Administrative Law* (Oxford, Oxford University Press 2012) ch 2. The legal origins of the precautionary principle are to be found in the *Vorsorgeprinzip* established by German environmental legislation in the mid-1970s; see, eg, T Christoforou, “The Origins and Content of the Precautionary Principle in European Community Law” in C Leben and J Verhoven (eds) *Le principe de precaution – Aspect de droit International et Communautaire* (Paris, Pantheon Assas LGDJ Diffuseur 2002). For the precautionary principle in health matters, see, eg, D Vogel, *The Politics of Precaution: Regulating Health, Safety, and Environmental Risks in Europe and the United States* (Princeton, NJ, Princeton University Press 2012).

41 In this article, I will refer only to the precautionary principle and not also to the prevention principle simply because the two principles are often discussed together and considered interchangeable in EU law. In fact, according to L Kramer, *EU Environmental Law* (London, Sweet & Maxwell 2016) p 24, there is a close relationship between the two principles that has led some to argue that they may be used “interchangeably”. However, other authors contend that the prevention principle applies in situations where the relevant risk is “quantifiable” or “known” and there is a certainty that damage will occur. In this sense, see, respectively, WT Douma, “The Precautionary Principle in the European Union” (2000) 9(2) Review of European Community & International Environmental Law 132; see also E Fisher, *Risk Regulation and Administrative Constitutionalism* (Oxford, Hart Publishing 2007) p 318. For an exhaustive explanation, see S Kingston, V Heyvaert and A Cavoski, *European Environmental Law* (Cambridge, Cambridge University Press 2017) p 99. By contrast, Italian legal doctrine makes a distinction between the precautionary principle and the prevention principle. Here, the mere potential or possibility of harm is the fundamental aspect whereby precaution differs from prevention, as the latter is founded in a situation of certainty. See, eg, F De Leonardis, *Il principio di precauzione nell’amministrazione del rischio* [*The Precautionary Principle in Risk Administration*] (Milan, Giuffrè 2005); A Barone, *Il diritto del rischio* [*The Law of Risk*] (Milan, Giuffrè 2006); see also F De Leonardis, “Principio di prevenzione e novità normative in materia di rifiuti” [Prevention Principle and New Legislation on Waste] (2001) 2 Rivista Quadrimestrale di Diritto Dell’ambiente 23–26. However, for the contention that combines precaution and prevention in the same principle, see, eg, P Savona, “Dal pericolo al rischio: l’anticipazione dell’intervento pubblico” [From Danger to Risk: The Anticipation of Public Intervention] (2010) 2 Diritto Amministravio 355.

42 ECJ Case T-13/99 *Pfizer Animal Health SA v Council* [2002] ECR II-3305; Cases T-74, 76, 83-85, 132, 137 and 141/00 *Artegodan GmbH v Commission* [2002] ECR II-4945.

43 ECJ Case C-77/09 *Gowan* [2010] ECR I-13533, paras 73–78. In the same sense, see also Case C-157/96 *National Farmers’ Union* [1998], ECR I-2211, para 63; Case C-180/96 *United Kingdom v. Commission* [1998], ECR I-2729, para 99; Case C-236/01 *Monsanto Agricoltura Italia* [2003], ECR II-8105, para 111.

44 Communication from the European Commission, On the Precautionary Principle, COM(2000)1 final, 8 <https://eur-lex.europa.eu/legal-content/EN/AUTO/?uri=celex:52000DC0001>.

45
ibid, 9-10.

46
ibid, 15.

47
ibid, 16–21.

48 In this sense, see the seminal work of CR Sunstein, *Laws of Fear. Beyond the Precautionary Principle* (Cambridge, Cambridge University Press 2005); CR Sunstein, “Beyond the Precautionary Principle” (2003) 151 University of Pennsylvania Law Review 1003 (from which I will quote). Sunstein enunciated a number of behavioural elements – such as loss aversion, probability neglect and neglect of the impact of one-off interventions – that might trigger people to blind themselves to certain aspects of the risk situation and focus on a particular subset of the hazards involved.

49
ibid, 1006, 1012–13 and 1023. The strong form of the principle imposes a burden of proof on those who create potential risks, and it requires regulation of activities even if it cannot be shown that those activities are likely to produce significant harms.

50
ibid, 1029.

51 A Vermeule, *The Constitution of Risk* (Cambridge, Cambridge University Press 2013). However, for some of the core concepts of Vermeule’s theory, see also A Vermeule, “Precautionary Principles in Constitutional Law” (2012) 4(1) Journal of Legal Analysis 181–222; A Vermeule, “Introduction: Political Risk and Public Law” (2012) 4(1) Journal of Legal Analysis 1–6. For the “mature” position terminology, Vermeule refers to AO Hirschman, *The Rhetoric of Reaction: Perversity, Futility, Jeopardy* (Cambridge and London, Belknap 1991).

52 Vermeule, *The Constitution of Risk*, supra, note 51, 3.

53
ibid, 49.

54
ibid, 71–72.

55
ibid, 52.

56
ibid, 24.

57 J Turley, “A Fox in the Hedges: Vermeule’s Vision of Optimized Constitutionalism in a Suboptimal World” (2015) 82 University Chicago Law Review 517–72; see also P Stone, “Review of *The Constitution of Risk*. By Adrian Vermeule. New York: Cambridge University Press, 2013. 200p.” (2015) 13(3) Perspectives on Politics 851–53.

58 Italian legal doctrine distinguishes between “administrative discretion” and “technical discretion” under the influence of MS Giannini, *Il Potere discrezionale della Pubblica Amministrazione* [*The Discretionary Power of the Administration*] (Milan, Giuffrè 1939) pp 74–79. On the difference, see E Presutti, “Discrezionalità pura e discrezionalità tecnica” [Discretion and Technical Discretion] (1910) 4 Giurisprudenza Italiana 15. See also F Ledda, “Potere, tecnica e sindacato giudiziario sull’amministrazione pubblica” [Power, Technical Assessments and Judicial Review of the Public Administration] (1983) 4 Diritto Processuale Amministrativo 371; C Marzuoli, *Potere amministrativo e valutazioni tecniche* [*Administrative Power and Technical Assessments*] (Milan, Giuffrè 1985); L Benvenuti, *La discrezionalità amministrativa*, [*Administrative Discretion*] (Padova, Cedam 1986); G Pastori, “Discrezionalità amministrativa e sindacato di legittimità” [Administrative Discretion and Judicial Review] (1987) Foro Italiano 3165. On discretion, in contemporary Italian scholarship, see D De Pretis, *Valutazioni amministrativa e discrezionalità tecnica* [*Administrative Assessments and Technical Discretion*] (Padova, Cedam 1995); A Police, *La predeterminazione delle decisioni amministrative. Gradualità e trasparenza nell’esercizio del potere discrezionale* [*The Predetermination of Administrative Decisions. Graduality and Transparency in the Exercise of Discretion*] (Napoli, ESI 1997); A Travi, “Il giudice amministrativo e le questioni tecnico-scientifiche: formule nuove e vecchie soluzioni” [The Administrative Judge and Technical-Scientific Problems: New and Old Solutions] (2004) 2 Diritto Pubblico 439; BG Mattarella, “Discrezionalità amministrativa” [*Administrative Discretion*] in S Cassese (ed), *Dizionario di diritto pubblico* (Milan, Giuffrè 2006) p 1993; F Volpe, “Discrezionalità tecnica e presupposti dell’atto amministrativo” [Technical Discretion and Prerequisites for the Administrative Act] (2008) 4 Diritto Amministravio 791; LR Perfetti, “Discrezionalità amministrativa, clausole generali e ordine giuridico della società” [Administrative Discretion, General Clauses and Legal Order of Society] (2013) 3 Diritto Amministravio 309. On the administrative discretion see the seminal book of D Galligan, *Discretionary Powers. A Legal Study of Official Discretion* (Oxford, Oxford University Press 1986) pp 1–55. Recently, see also J Bell, “Judicial Review in Administrative State” in J de Poorter, EH Ballin and S Lavrijssen (eds), *Judicial Review of Administrative Discretion in the Administrative State* (The Hague, Springer 2019) pp 3–27.

59 Art 77 of the Italian Constitution <https://www.senato.it/documenti/repository/istituzione/costituzione.pdf>.

60 See, eg, V Barsotti, PG Carrozza, M Cartabia and A Simoncini (eds), *Italian Constitutional Justice in Global Context* (Oxford, Oxford University Press 2016) pp 164–66.

61 G Berti and GC De Martin (eds), *Il sistema amministrativo dopo la riforma del Titolo V della Costituzione* [*The Adminsitrative System after the Reform of Title V*] (Rome, Luiss Press 2002).

62 S Cassese, “L’amministrazione nel nuovo Titolo V della Costituzione” [The Administration in the New Title V of the Constitution] (2011) Giornale di Diritto Amministrativo 1193.

63 G Berti, “Amministrazione e costituzione” [Administration and Constitution] (1993) 1 Diritto Amministrativo 455.

64 G Pastori, “Sussidiarietà e diritto alla salute” [Subsidiarity and Right to Health] (2002) 1 Diritto Pubblico 89.

65 The legal nature of the “State’s substitution power” in Italian legal doctrine has been extensively discussed. In particular, some scholars argue that Art 120 provides a form of “administrative” substitution of the State over the Regions, and that Art 117(5) concerns “legislative” substitution. Other scholars agree on the idea that Art 120 provides the *genus* of substitution powers, whereas Art 117(5) refers to one *species* of the *genus*, being a mere specification of Art 120. However, the Constitution seems clear on this point. As we have seen, the provisions of Art 120 speak of the “Government”, while the provisions of Art 117(5) speak of the “State”. For a reconstruction of the debate in Italian legal doctrine, see C Mainardis, *Poteri sostitutivi statali e autonomia amministrativa regionale* [*State Substitute Powers and Regional Administrative Autonomy*] (Milan, Giuffrè 2007) pp 5–14.

66 For an analysis of emergency administrative powers in the Italian legal system, see E Casetta, *Manuale di diritto amministrativo* [*Handbook of Administrative Law*] (Milan, Giuffrè 2018) pp 375–79.

67 OECD, “The Territorial Impact of COVID-19: Managing the Crisis across Levels of Government” (last updated 16 June 2020) <https://read.oecd-ilibrary.org/view/?ref=128_128287-5agkkojaaa&title=The-territorial-impact-of-covid-19managing-the-crisis-across-levels-of-government>.

68 The European Committee of the Regions (CoR), “Division of Powers between the European Union, the Member States and Regional and Local Authorities” (December 2012) <https://cor.europa.eu/en/engage/studies/Documents/Study-Division-Powers/Study-Division-Powers-EN.pdf>. See also, OECD-PUMA, “Managing across Levels of Government” (1997) <http://www.oecd.org/governance/budgeting/1902308.pdf>.

69 On this approach, see G Pisano, R Sadum and M Zanini, “Lessons from Italy’s Response to Coronavirus” (Harvard Business Law Review, 27 March 2020) <https://hbr.org/2020/03/lessons-from-italys-response-to-coronavirus#comment-section>.

70 DPCM of 9 March 2020 <www.gazzettaufficiale.it/eli/gu/2020/03/09/62/sg/pdf>.

71 See, eg, R Li et al, “Substantial Undocumented Infection Facilitates the Rapid Dissemination of Novel Coronavirus (SARS-CoV2)” (2020) 368 Science 489–93.

72 See, eg, VJ Munster et al, “A Novel Coronavirus Emerging in China, Key Questions for Impact Assessment”, (2020) 382 New England Journal of Medicie 692–94.

73 C Zhu and J Zhu, “Spread Trend of COVID-19 Epidemic Outbreak in China” (2020) 17(4) Mathematical Biosciences and Engineering 3062–87. For daily reports around the world, cf. WHO, “Coronavirus Disease (COVID-2019) Situation Reports” <https://www.who.int/emergencies/diseases/novel-coronavirus-2019/situation-reports/>.

74 For an analysis of other paradigmatic cases, see Simoncini, supra, note 10.

75 Decree No. 56 of 27 February 2020 of the Marche Regional Administrative Court suspended the Marche Region Ordinance issued on 25 February 2020. For a comment on this judgment, see G di Cosimo and G. Menegus, “La gestione dell’emergenza coronavirus tra stato e regioni: il caso Marche” [Coronavirus Emergency Management between State and Regions: the Case of the Marche Region] (2020) 2 BioLaw Journal 1–7.

76 The Campania Regional Administrative Court, Section V, Decree No. 416 of 18 March 2020, relating to Campania Region Ordinance No. 15 of 13 March 2020.

77 G Giordano et al, “Modelling the COVID-19 Epidemic and Implementation of Population-Wide Interventions in Italy” (2020) 26 Nature 855–60.

78 Regarding the coordination of powers, see Nicola, supra, note 7; Simoncini, supra, note 10.

79 See, eg, Y Bai et al, “Presumed Asymptomatic Carrier Transmission of COVID-19” (2020) 323(14) JAMA 1406–07.

80 ZA Nasir et al, “Airborne Biological Hazards and Urban Transport Infrastructure: Current Challenges and Future Directions” (2016) 23(15) Environmental Science and Pollution Research 15757–66.

81 L Setti et al, “Evaluation of the Potential Relationship between Particulate Matter (PM) Pollution and COVID-19 Infection Spread in Italy” (2020) Italian Society of Environmental Medicine <http://www.simaonlus.it/wpsima/wp-content/uploads/2020/03/COVID_19_position-paper_ENG.pdf>.

82 See, eg, F Cortese, “Stato e Regioni alla prova del coronavirus” [State and Regions Put to the Test by Coronavirus] (2020) 1 Le Regioni 4–10.

83 See Nicola, supra, note 7.

84 CM Weible et al, “COVID-19 and the Policy Sciences: Initial Reactions and Perspectives” (2020) 53 Policy Science 225–41.

85
ibid, 231.

86
ibid, 231.

87 P Cairney, *The Politics of Evidence-Based Policymaking* (London, Palgrave Macmillan 2016).

88 Weible et al, supra, note 84, 231.

89
ibid, 231.

90 A Alemanno, “The European Response to COVID-19: From Regulatory Emulation to Regulatory Coordination?” (2020) 11(2) European Journal of Risk Regulation 307–16.

91 WHO, “Pandemic Influenza Risk Management Guidance” (17 May 2017) <https://www.who.int/influenza/preparedness/pandemic/PIRM_update_052017.pdf>.

92 Li et al, supra, note 71, 1–8.

93 For the legal structure of the Italian health care system, see, eg, A Pioggia, *Diritto sanitario* [*Health Law*] (Turin, Giappichelli 2017) p 77.

94 A Rhodes et al, “The variability of critical care bed numbers in Europe” (2012) 38 Intensive Care Medicine 1647–53.

95 WHO, “Pandemic Influenza Risk Management Guidance” (May 2017) <https://www.who.int/influenza/preparedness/pandemic/PIRM_update_052017.pdf>.

96 The WHO’s Guidelines on the “Pandemic Influenza Risk Management” update and supersede “Pandemic influenza preparedness and response: a WHO Guidance document”, which was published in 2009. The WHO’s Guidelines of 2017 take account of lessons learnt from the influenza A(H1N1) 2009 pandemic and other significant developments.

97 National Plan for Preparedness and Response to an Influenza Pandemic, Ministry of Health, 13 December 2007 <http://www.salute.gov.it/imgs/C_17_pubblicazioni_511_allegato.pdf>.

98 The failure to implement the pandemic influenza preparedness plan triggered a parliamentary question by a member of the Senate, Mr Gregorio de Falco. In this regard, see Act No. 3-01464 (with Emergency Procedures), 1 April 2020, session no. 204 <http://www.senato.it/japp/bgt/showdoc/showText?tipodoc=Sindisp&leg=18&id=1148825>.

99 Legislative Decree 303/1999 <http://www.parlamento.it/parlam/leggi/deleghe/99303dl.htm>.

100 Italy’s national pandemic plan was adopted through the Permanent Conference on the Relationships between Central Government, the Regions, Municipalities and other Local Authorities <http://www.regioni.it/sanita/2006/02/14/accordo-stato-regioni-9-2-06-su-pandemia-influenzale-97179/>.

101 Law No 267/2000 <https://www.gazzettaufficiale.it/eli/id/2000/09/28/000G0304/sg>.

102 Law No 400/1988 <https://www.gazzettaufficiale.it/eli/id/1988/09/12/088G0458/sg>.

103 Recently, see Council of State, Section I, 7 April 2020, No. 735.

